# Chikungunya-Associated Acute Respiratory Distress Syndrome: A Rare Fatal Case

**DOI:** 10.7759/cureus.115063

**Published:** 2026-08-24

**Authors:** Arkaprava Singharoy, Payel Bose, Tanmay Banerjee, Sagnik Das, Prerona Bhowmick

**Affiliations:** 1 Department of Critical Care Medicine, Manipal Hospital, Kolkata, IND

**Keywords:** acute respiratory distress syndrome, atypical chikungunya, chikungunya infection, critical care, tropical infection

## Abstract

Chikungunya fever is an arboviral disease caused by an alphavirus belonging to the family Togaviridae. It commonly presents with fever, severe arthralgia, rash, and myalgia, while severe pulmonary involvement causing acute respiratory distress syndrome (ARDS) is rare. We report a 62-year-old man with obesity, diabetes mellitus, hypertension, obstructive sleep apnoea, coronary artery disease, and interstitial lung disease, who presented with high-grade fever, severe arthralgia, facial puffiness, and rapidly progressive dyspnoea, following recent travel to Lucknow, India. On admission, he had severe hypoxemia with a PaO₂/FiO₂ ratio below 100, requiring mechanical ventilation. During intubation, he developed cardiac arrest but achieved return of spontaneous circulation after cardiopulmonary resuscitation. Chikungunya IgM and reverse transcriptase polymerase chain reaction (RT-PCR) were positive. Chest imaging showed diffuse bilateral infiltrates and ground-glass opacities consistent with severe ARDS. Dengue, malaria, and scrub typhus were excluded. The patient was managed with lung-protective ventilation, prone positioning, and corticosteroids according to the Meduri protocol with transient improvement. However, he later developed worsening respiratory failure and multiorgan dysfunction and eventually succumbed after 41 days of hospitalization. Although chikungunya is not typically considered a respiratory virus, clinicians should recognize that severe pulmonary manifestations, including ARDS, may occur, particularly in elderly patients with significant comorbidities.

## Introduction

Chikungunya fever is an arboviral disease caused by an alphavirus belonging to the family Togaviridae. Historical descriptions of epidemics characterized by fever and severe arthralgia date back to the 18th century, while the first officially documented outbreak occurred in 1952 on the Makonde Plateau in Africa [1]. In India, outbreaks were reported between 1963 and 1973, followed by a prolonged period of relative quiescence until its re-emergence in 2005, initially in southern India and subsequently across multiple regions of the country [2].

Typical clinical manifestations include fever, polyarthralgia, rash, headache, and lymphadenopathy. While most cases are self-limiting, atypical and severe manifestations involving the neurological, cardiovascular, renal, hepatic, and respiratory systems have increasingly been recognized [3].

Acute respiratory distress syndrome (ARDS) is a life-threatening form of acute hypoxemic respiratory failure and a common complication of severe viral pneumonia requiring intensive care. Several respiratory viruses, including influenza viruses, respiratory syncytial virus, rhinoviruses, and coronaviruses, are well-recognized causes of viral pneumonia-associated ARDS [4].

Pulmonary involvement in chikungunya infection is uncommon, while severe pulmonary complications such as respiratory failure and ARDS are rare and have been reported predominantly in severe or atypical cases [3,5]. ARDS secondary to chikungunya infection carries significant morbidity and mortality and remains poorly described in the literature. We present a case of severe chikungunya infection complicated by ARDS in a patient with multiple comorbidities, highlighting the clinical course, management challenges, and outcome.

## Case presentation

**Figure 1:**
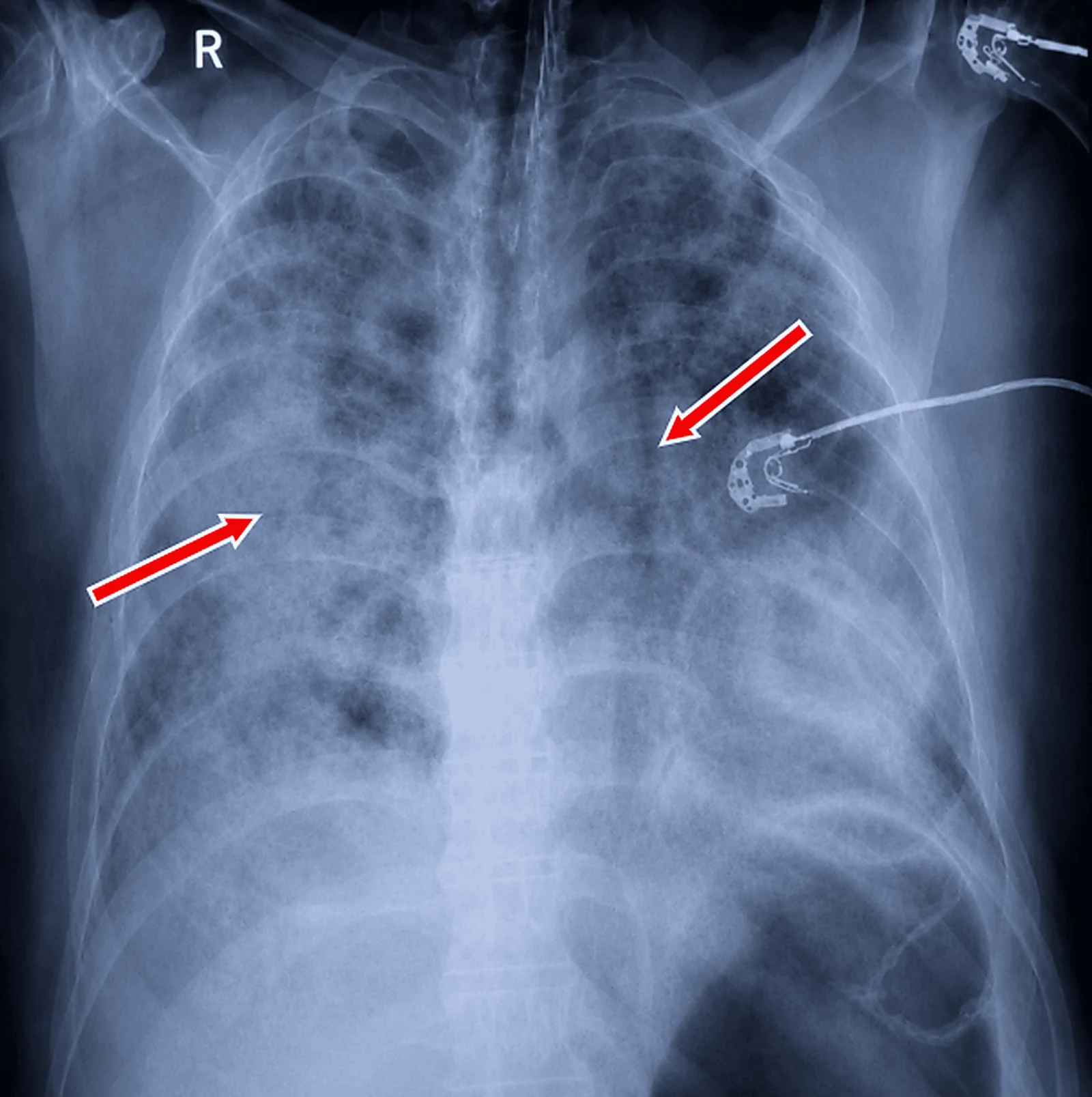
Chest radiography showing loss of aeration (arrows) in almost all lung fields due to infiltrates

**Figure 2:**
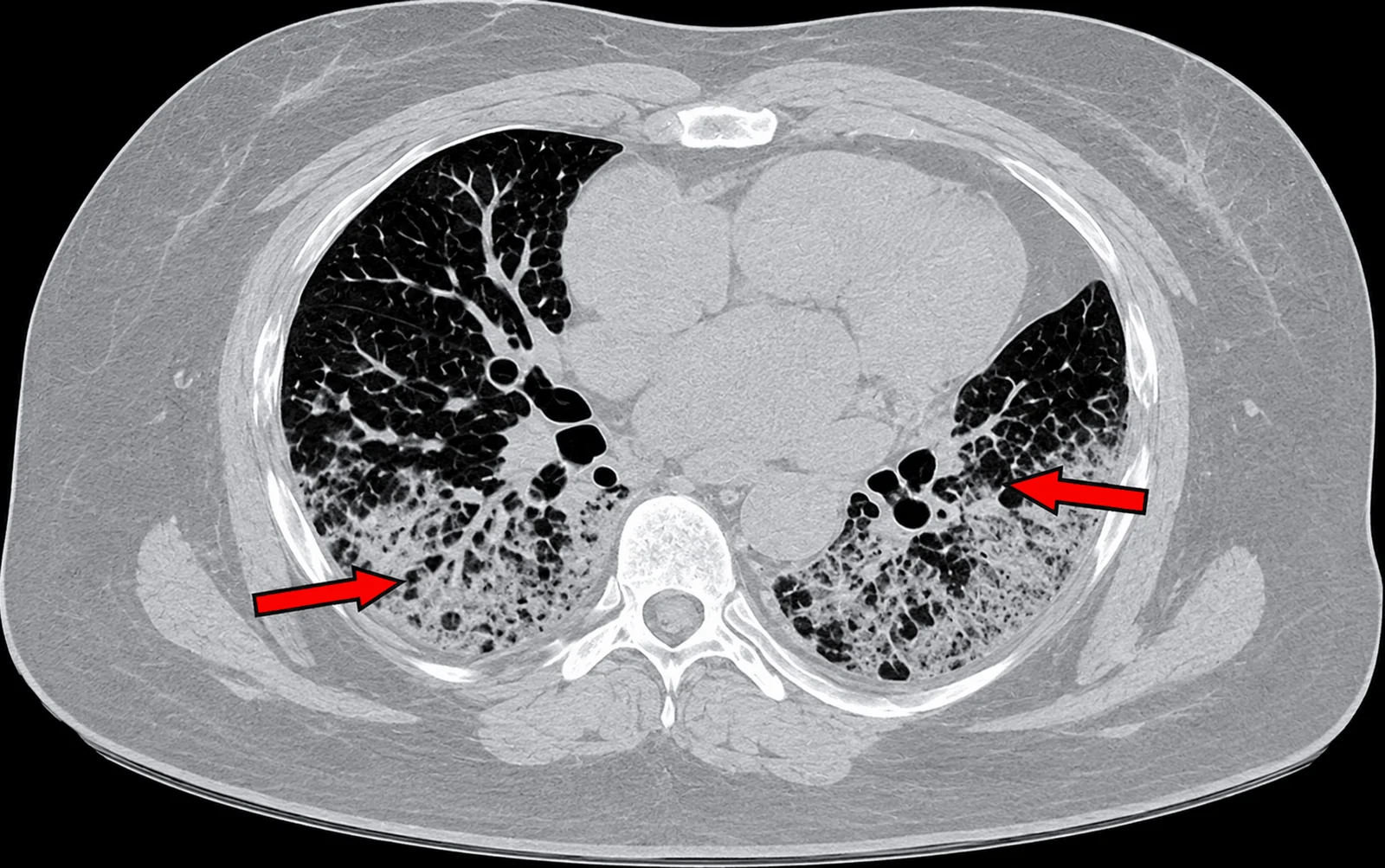
High-resolution computed tomography (HRCT) of the chest showing opacities with underlying bronchiectasis and thickened interlobular septum (arrows) involving both lungs

**Figure 3:**
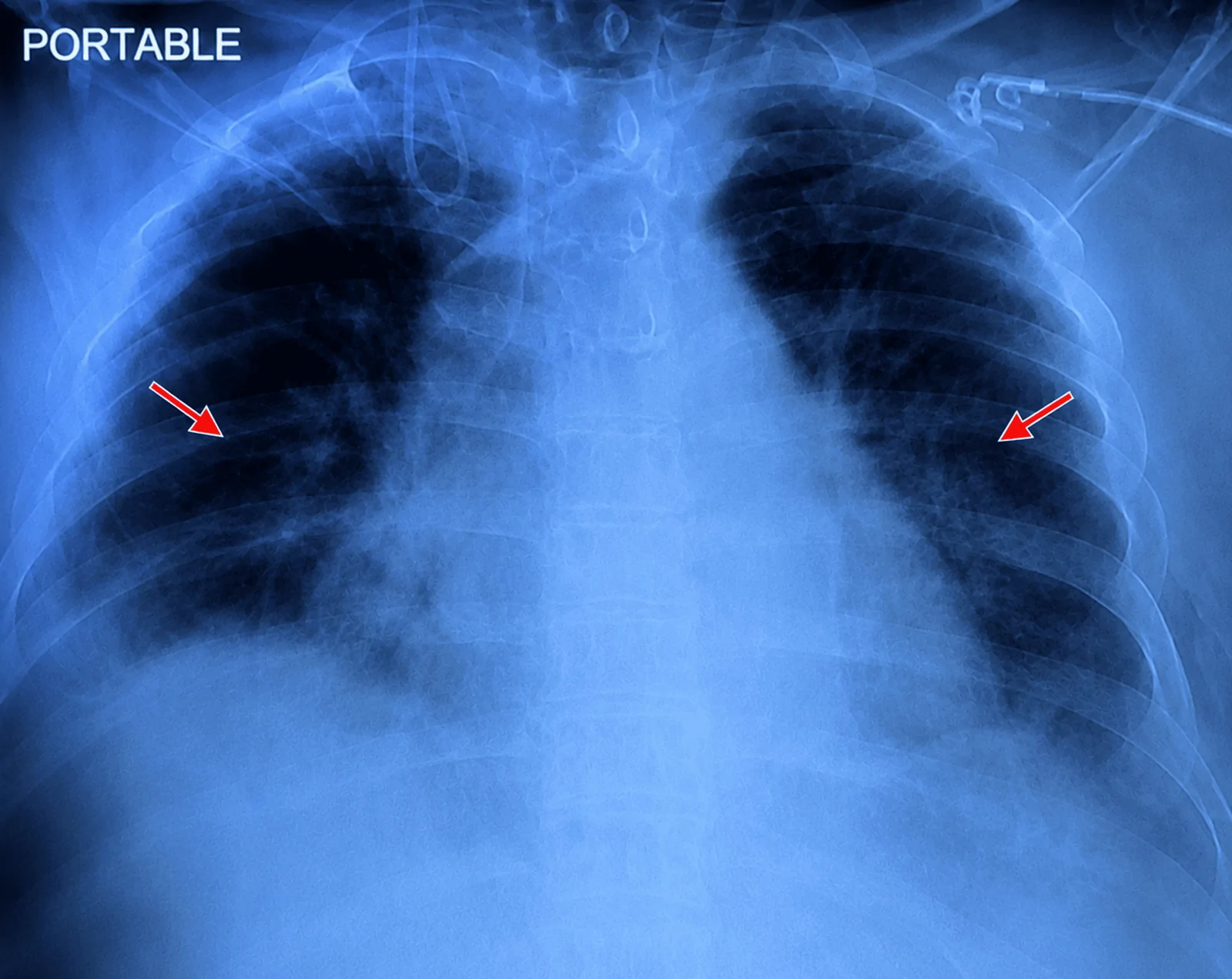
Improvement in chest radiography following steroid therapy

**Figure 4:**
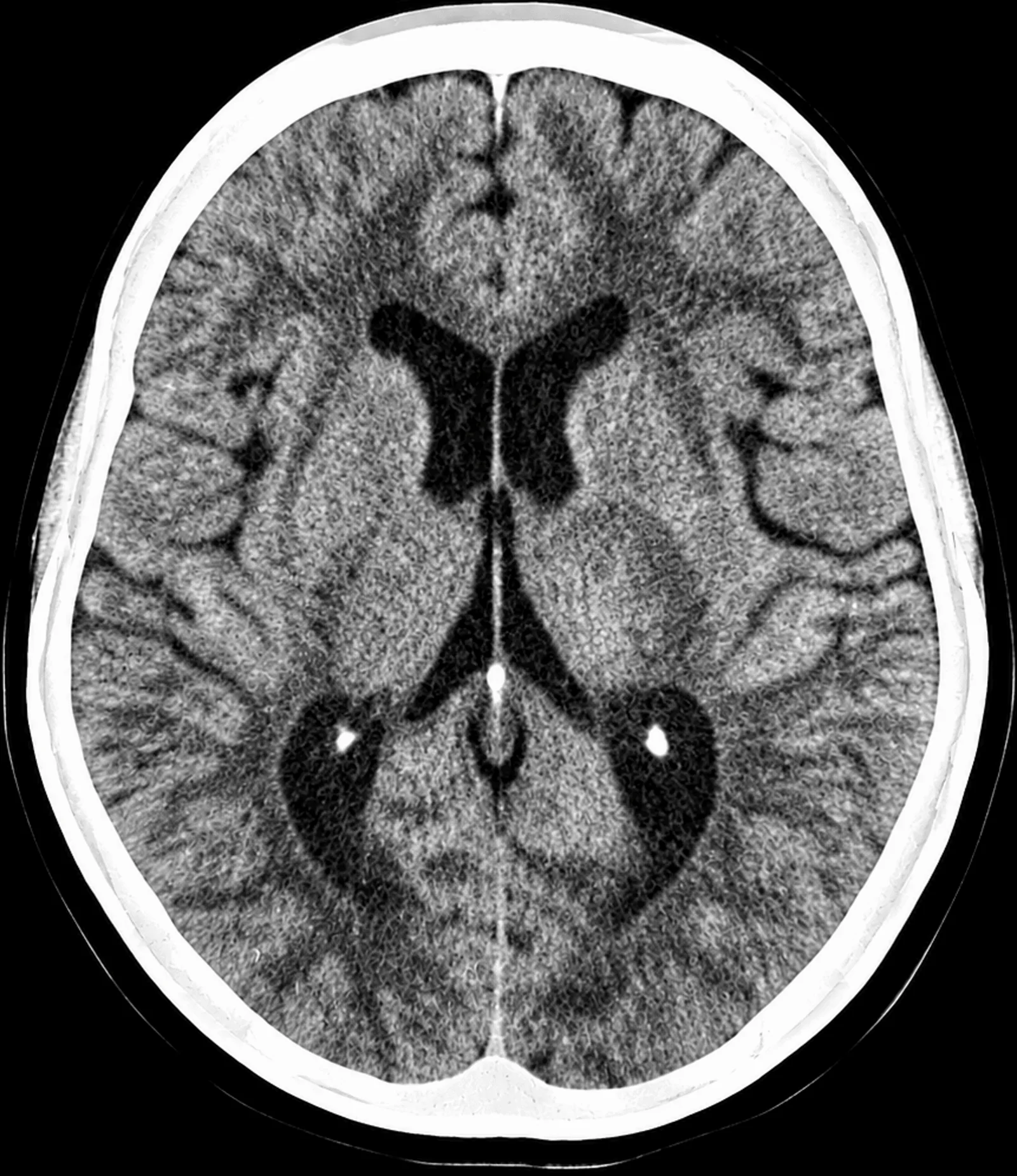
Normal CT brain when the patient developed myoclonus

**Figure 5:**
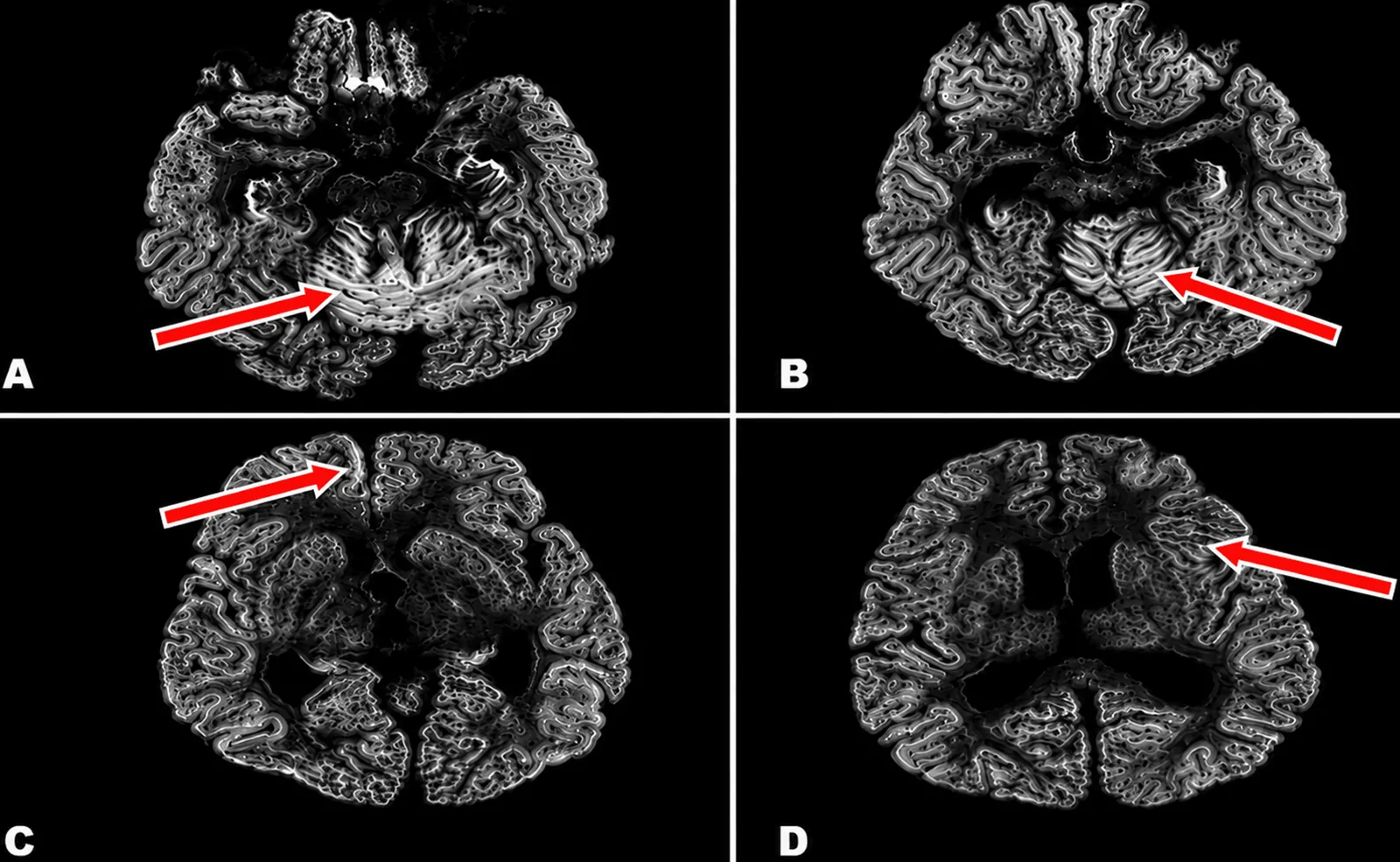
Axial brain MRI at different levels demonstrating hypoxic-ischemic encephalopathy (A) Cerebellar cortex. (B) Thalami and deep gray nuclei. (C) Frontal cortex. (D) Parietal cortex. Arrows indicate the areas of abnormal signal intensity.

**Figure 6:**
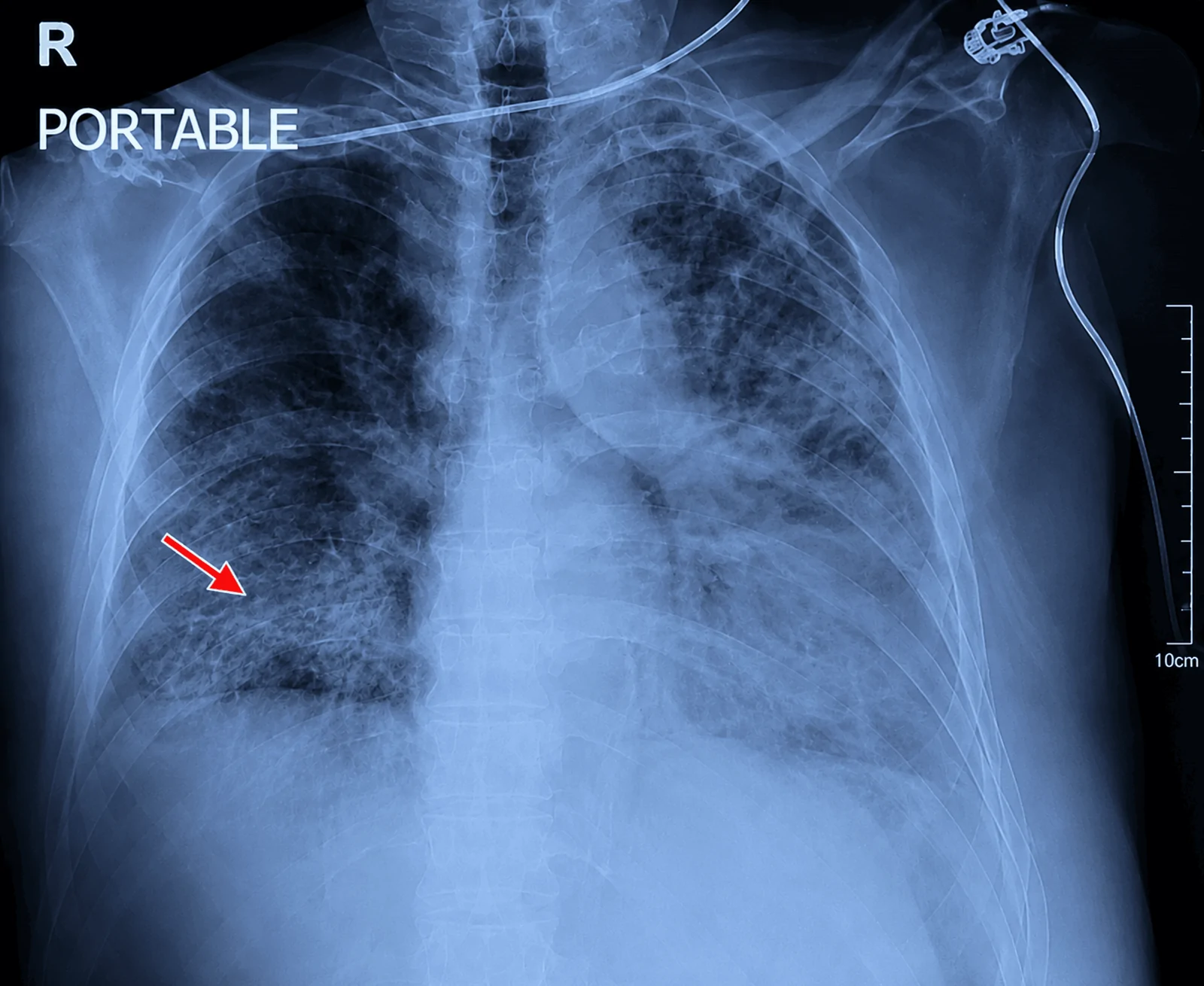
Repeat chest radiography showing new consolidation (arrow)

**Figure 7:**
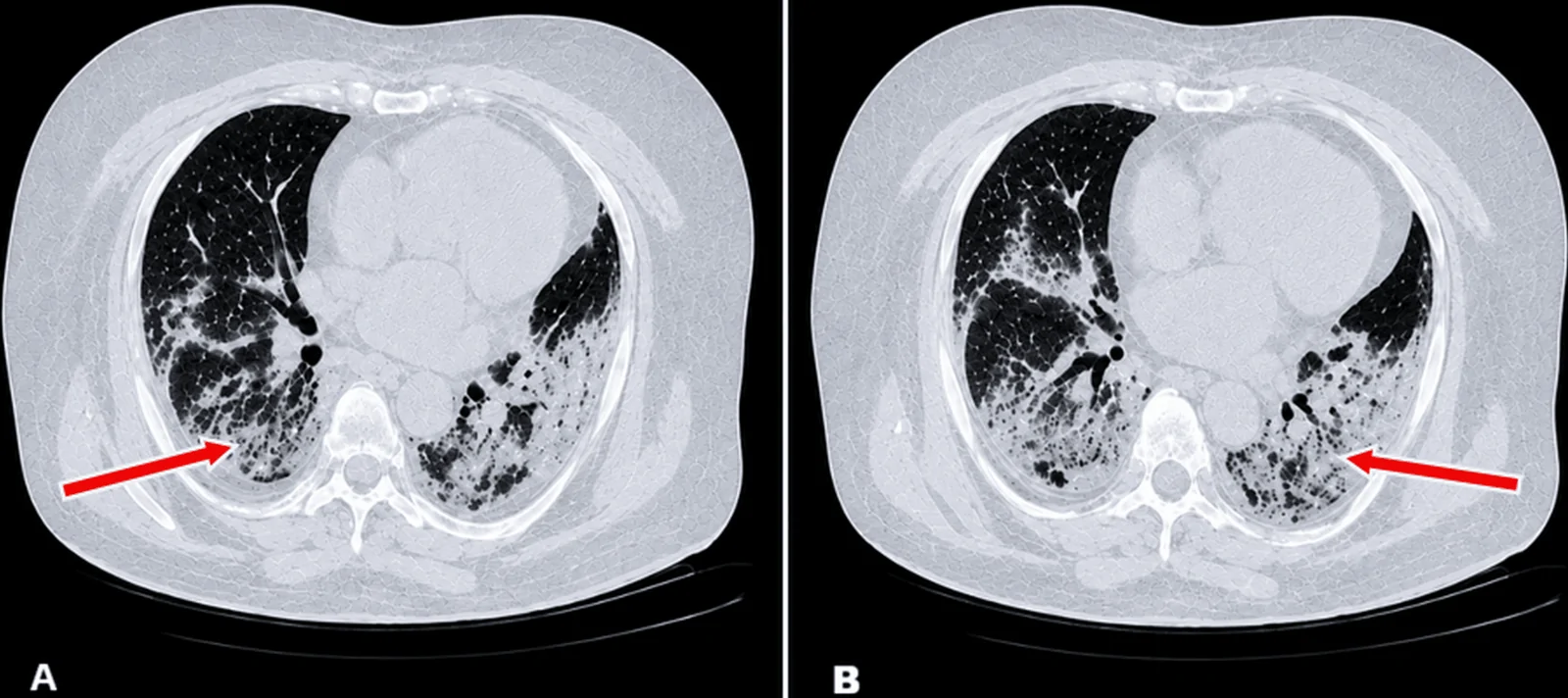
Repeat high-resolution computed tomography (HRCT) of chest showing extensive progression of lung injury Axial HRCT images obtained at different levels demonstrate bilateral diffuse ground-glass opacities with patchy consolidation and traction bronchiectatic changes (arrows), consistent with progressive diffuse lung injury.

A 62-year-old man presented with high-grade fever for four days associated with severe arthralgia, facial puffiness, redness, and acute-onset progressive breathlessness for five to six hours. He had travelled to Lucknow, India, one week prior to symptom onset. His medical history included morbid obesity, hypertension, type 2 diabetes mellitus, obstructive sleep apnoea, coronary artery disease, and interstitial lung disease. On admission, the patient was severely hypoxemic with a PaO₂/FiO₂ ratio below 100 and required urgent intubation and invasive mechanical ventilation. During intubation, he developed cardiac arrest and underwent cardiopulmonary resuscitation for eight minutes before achieving return of spontaneous circulation. Given the presentation of fever and severe arthralgia, tropical infectious diseases were investigated. Chikungunya IgM serology and serum reverse transcriptase polymerase chain reaction (RT-PCR) were positive. Dengue, malaria, and scrub typhus testing were negative. A respiratory viral panel, including influenza A and B, SARS-CoV-2, respiratory syncytial virus (RSV) A/B, parainfluenza viruses 1-4, adenovirus, and rhinovirus, was performed and was negative for all tested pathogens. Chest radiography revealed absolute loss of aeration in almost all lung fields due to infiltrates (Figure 1). High-resolution computed tomography (HRCT) demonstrated bilateral consolidations and diffuse ground-glass opacities (Figure 2). Based on the Berlin criteria, he was diagnosed with severe ARDS [6]. The patient was managed with lung-protective ventilation strategies and prone positioning [7]. The patient developed acute kidney injury with oliguria but responded to furosemide challenge, and his oxygenation improved upon proning. The following day, the patient developed myoclonus and was treated with intravenous levetiracetam and midazolam. CT brain was done, which was normal (Figure 3). Intravenous corticosteroid infusion was initiated, considering the severe inflammatory lung injury and refractory hypoxemia, according to the Meduri protocol for ARDS [8]. Significant improvement in oxygenation and chest radiographic findings was observed following steroid therapy (Figure 4). Magnetic resonance imaging of the brain later demonstrated hypoxic-ischemic encephalopathy, likely secondary to the cardiac arrest episode (Figure 5). On Day 11, the patient developed worsening oxygen requirements, accompanied by rising total leukocyte count, procalcitonin, and C-reactive protein levels. Blood and bronchoalveolar lavage culture remained sterile throughout the hospital course, with no microbiological evidence of a concomitant bacterial or fungal infection. Repeat chest radiography demonstrated new pulmonary consolidations (Figure 6). Steroid infusion was discontinued, and hydrocortisone bolus therapy was initiated. Following this, the patient developed progressive multiorgan dysfunction and worsening pulmonary compliance. Repeat HRCT imaging demonstrated extensive progression of lung injury (Figure 7). The patient finally succumbed to his illness after 41 days of hospitalization.

## Discussion

Chikungunya infection is usually self-limiting; however, severe disease can occur in elderly patients, neonates, and those with significant comorbidities [9]. Our patient had multiple risk factors associated with poor outcomes, including obesity, diabetes mellitus, underlying interstitial lung disease, and cardiovascular disease. The chikungunya virus exhibits broad cellular tropism involving endothelial cells, epithelial cells, and fibroblasts, leading to initial activation of innate immune responses and also dissemination of the virus through various lymphoid tissues to other organs [9]. In the acute phase, there is release of various type 1 interferons, as well as upregulation and downregulation of other cytokines [3,9]. Although musculoskeletal involvement remains the hallmark of chikungunya infection, atypical manifestations, such as myocarditis, hepatitis, nephritis, meningoencephalitis, and Guillain-Barré syndrome, have been documented [9]. The viral disease can progress to a chronic phase with prolonged infection of the synovial tissues and muscle cells, as it may survive in such areas of the body that may evade host immune surveillance [9]. Excessive inflammatory responses are believed to contribute to diffuse alveolar damage and ARDS. Pulmonary complications, particularly ARDS, are rare, with only a few reported cases in the literature [5], but are associated with high mortality rates approaching 46% in some reports [3].

Previous reports by Singh et al. and Khare et al. described favourable outcomes in chikungunya-associated ARDS with supportive care and corticosteroid therapy [5,10]. Similarly, our patient initially demonstrated improvement after corticosteroid administration according to the Meduri protocol, suggesting a possible beneficial role of immunomodulation in selected patients with severe inflammatory lung disease. However, the presence of severe comorbidities and hypoxic brain injury likely contributed to the poor outcome.

## Conclusions

Chikungunya fever can rarely present with severe pulmonary involvement leading to ARDS and multiorgan dysfunction. This case emphasizes that respiratory manifestations should not exclude chikungunya from the differential diagnosis in endemic settings, particularly when accompanied by fever and severe arthralgia during outbreaks or following travel exposure. Early recognition of atypical respiratory manifestations, prompt intensive care support, and timely initiation of appropriate therapies may improve outcomes.
